# TYPOLOGIES OF SOURCE OF CARE AMONG OLDER ADULTS: A COMPARATIVE STUDY OF CHINA AND THE UNITED STATES

**DOI:** 10.1093/geroni/igad104.3057

**Published:** 2023-12-21

**Authors:** Dexia Kong, Peiyi Lu

**Affiliations:** The Chinese University of Hong Kong, Hong Kong, Hong Kong; Columbia University, New York City, New York, United States

## Abstract

**Background:**

We investigated typologies of source of care for older adults in China and the United States and applied Anderson Health Behavior model to examine associated significant determinants.

**Methods:**

Cross-sectional data from 2014 Health and Retirement Study and 2013 China Health and Retirement Longitudinal Study were used. We included older respondents aged 65+ who had at least one limitation in activities of daily living (ADL) and instrumental ADL (IADL) (NChina=2,482, NUS=3,152). Respondents reported whether they received assistance with ADLs and IADLs from spouse, child/grandchild, relatives, other, and formal helpers, respectively. We employed latent class analysis to characterize the typologies of sources of care and multinomial logistic regression to examine the significant determinants of identified typologies.

**Results:**

Four classes were identified in China: class 1 (59.91%) minimal care with IADL assistance from child/grandchild; class 2 (8.66%) child/grandchild-based care; class 3 (27.96%) spouse-based care; and class 4 (3.46%) spouse/child/grandchild-based care. Five classes were identified in the US: class 1 (51.97%) minimal care overall with limited spousal support; class 2 (20.88%) child/grandchild-based care; class 3 (8.44%) spouse-based care; class 4 (13.20%) formal care plus child/grandchild support; and class 5 (6.31%) various sources. In both countries, ADL and IADL were significant determinants.

**Conclusion:**

In the US, sources of care were more diverse and included formal assistance. In contrast, older Chinese relied largely on their spouses and children/grandchildren for support. In both countries, physical circumstances are significant determinants. Policy efforts on supporting family-based care and expanding formal care are needed in both countries, particularly China.

